# Effects of Nocturnal Aircraft Noise and Non-Acoustical Factors on Short-Term Annoyance in Primary School Children

**DOI:** 10.3390/ijerph18136959

**Published:** 2021-06-29

**Authors:** Julia Quehl, Susanne Bartels, Rolf Fimmers, Daniel Aeschbach

**Affiliations:** 1 Department of Sleep and Human Factors Research, Institute of Aerospace Medicine, German Aerospace Center (DLR), 51147 Cologne, Germany; julia.quehl@dlr.de (J.Q.); daniel.aeschbach@dlr.de (D.A.); 2 Department of Medical Biometry, Informatics and Epidemiology (IMBIE), Faculty of Medicine, University of Bonn, 53127 Bonn, Germany; fimmers@ukb.uni-bonn.de; 3 Faculty of Medicine, University of Bonn, 53127 Bonn, Germany

**Keywords:** nocturnal aircraft noise, children, vulnerable groups, annoyance, exposure–response models, non-acoustical factors

## Abstract

Children are considered at higher risk for harmful noise effects due to their sensitive development phase. Here, we investigated the effects of nocturnal aircraft noise exposure on short-term annoyance assessed in the morning in 51 primary school children (8–10 years) living in the surrounding community of Cologne-Bonn Airport. Child-appropriate short-term annoyance assessments and associated non-acoustical variables were surveyed. Nocturnal aircraft noise exposure was recorded inside the children’s bedrooms. Exposure–response models were calculated by using random effects logistic regression models. The present data were compared with those from a previous study near Cologne-Bonn Airport in adults using very similar methodology. Short-term annoyance reaction in children was not affected by the nocturnal aircraft noise exposure. Non-acoustical factors (e.g., the attitude that “aircraft are dangerous” or noise sensitivity), however, significantly impacted on children’s short-term annoyance. In contrast to children, the probability of moderate to high annoyance in adults increased with the number of aircraft flyovers during the time in bed. It is concluded that short-term annoyance from nocturnal aircraft noise in children is mainly determined by non-acoustical factors. Unlike in adults, acoustical factors did not play a significant role.

## 1. Introduction

Rising demand for air, rail, and road travel implies that a growing number of people is being exposed to environmental noise, and noise exposure is increasingly being regarded as a prominent public health problem [1,2]. Noise is a main cause of environmental annoyance, and it negatively influences quality of life for large parts of the population. Environmental noise is generally defined as any kind of unwanted sound resulting from human activities, including noise emitted by all modes of transport [3]. Negative effects of traffic noise exposure on adults with respect to cardiovascular diseases [4,5,6,7,8] mental health [9,10,11,12], annoyance [5,13,14], cognitive performance and communication [5,15], and sleep disturbances [4,5,16,17,18] are empirically very well-documented. With regard to the number of affected residents, sleep disturbances manifested in changes in sleep depth and sleep continuity, awakening reactions and reduced (self-rated) sleep quality are considered to be the most serious impact of environmental noise [19,20,21].

Recently, noise effects research has increasingly focused on “groups at risk” or “susceptible groups” [22], i.e., the parts of the population who show particular vulnerability to harmful noise impacts. These so-called vulnerable groups include elderly and sick people, shift workers, as well as children [5]. It can be assumed that the majority of children living in urbanized regions are more or less affected by traffic noise throughout the day. Negative effects of chronic noise exposure on children’s learning and cognitive performance [5,12,15,23,24,25,26,27,28,29,30,31,32,33,34,35,36], motivation [24,37,38], and quality of life including annoyance [15,24,28,30,31,39,40,41,42,43,44] have been extensively described in the literature so far. Only few well-founded studies on noise impacts on sleep in children have been available up to now [45,46,47] even though their sleep is expected to be particularly sensitive to traffic noise impacts [48,49,50,51]. Different physiological and psychological approaches have been used to explain the adverse effects of noise exposure on children [12,24,25,39,41,52,53,54,55,56,57,58]. An example is the theory of environmental stress [27,59]. The central aspect of this approach assumes that noise represents an environmental stressor, i.e., persons living in noisy environments see noise as a threat and perceive it stressful. This supposition is particularly important given that children’s ability to estimate or judge threats from environmental stressors is not fully developed. Thus, they have less control over threatening situations [60] which may result in increased susceptibility to adverse noise impacts.

Annoyance is the primary psychological parameter used to describe the impact of environmental noise on populations [61]. Annoyance primarily follows the noise-induced disturbance of (intended) activities [3] and includes negative emotional reactions to noise events [39,62]. Activities such as communication, relaxation, and recuperation are considered to be especially sensitive to noise-induced disturbances [3,61,62,63]. Sleep disturbances are the most common reason for noise complaints [64,65,66]. It is assumed that children’s and adults’ noise annoyance may be explained on the basis of the same psychological construct given that the emotional responses to disturbing noise events are similar [27,60]. Only a few studies have developed exposure–response models for noise-induced annoyance in children. In the cross-sectional EU project RANCH (Road traffic noise and Aircraft Noise exposure and children’s Cognition and Health), a curvilinear exposure–response relationship with chronic aircraft noise exposure both at school and at home was found for annoyance response in children (aged 9–11 years) when adjusted for confounding variables [42,67,68,69,70]. This model showed that children chronically exposed to aircraft noise had a significantly higher noise annoyance than children attending low aircraft noise exposed in both settings. Van Kempen et al. [70] documented that the exposure–response curves for aircraft noise annoyance in children and their parents were similar. However, children scored lower on annoyance at the top of the scale, and slightly higher at the lower end. Further studies on children’s long-term annoyance from chronic road [4] and aircraft noise [22,42,67,68,69,70] exposure indicated that children were noticeably less annoyed by traffic noise exposure than adults.

An established rule of thumb in noise effects research is that about one third of annoyance in adults is due to acoustical variables, another third caused by non-acoustical factors, while the last third has not been specified so far [3,13,39,63,71,72,73,74,75,76,77]. The individual noise sensitivity is an important personal variable [18,72,73,77,78,79]. There are a variety of psychological factors related to residents’ views on air transport [13,77,80,81,82,83]. They include, for instance, residents’ beliefs about how air traffic may affect them (including perceived social and economic advantages/disadvantages and its evaluation regarding the harm to one’s health), attitudes towards air traffic, feelings of helplessness in controlling air traffic and aircraft noise exposure, negative expectations regarding future aircraft noise development, perceived fairness and consideration of local residents’ interests in decision-making processes of the airport management, adaptation to long-term aircraft noise exposure, and worries about safety (e.g., fear of aircraft crashes). Though, studies examining the impact of non-acoustical influence variables with respect to children’s annoyance are sparse (e.g., [39,84]).

Recently, we investigated the acute physiological effects of night-time aircraft noise on sleep and associated psychological reactions (i.e., short-term annoyance, self-rated sleep quality, and cognitive performance) of primary school children. Results of the aircraft noise-induced short-term annoyance during the past night in children are presented here. A new children’s annoyance model was developed. For adult’s annoyance, the influence of acoustical and non-acoustical parameters has been widely demonstrated [13,39,71,72,73,75,76,77,80,81,83]. As noise-induced annoyance response in children is based on the same psychological construct used in adults [27,60], it was hypothesized that both acoustical and non-acoustical factors contribute to children’s short-term noise annoyance. Exposure–response models for annoyance due to night-time aircraft noise exposure were calculated by means of random effects logistic regression. The selection of potential acoustical and psychological predictors was based on previous studies on (aircraft) noise impact on annoyance in adults. To allow for a cross-sectional comparison of aircraft noise annoyance in children and adults, we also included data from a previous study (STRAIN; Study on Human Specific Response to Aircraft Noise) conducted by the German Aerospace Center (DLR) near Cologne-Bonn Airport in 2001/2002 in 64 adult residents. Data from the present and the STRAIN study [85] were compared on the basis of exposure–response models in order to reveal potential differences in the annoyance assessments between children and adults.

## 2. Materials and Methods

### 2.1. Participants

Fifty-one healthy and normal-hearing children aged 8 to 10 years participated in the study. There were 23 females (45%). All children passed a multi-stage selection procedure including questionnaires screening for major medical or intrinsic sleep disorders as well as an audiometric screening. Both children and parents volunteered to participate in the study and gave written informed consent in accordance with the guidelines of the Declaration of Helsinki prior to the study. The study protocol was approved by the ethics board of the North Rhine Chamber of Physicians. Participating children received age-adequate remuneration in the form of a voucher for a toy online shop or an amusement park. Parents received EUR 50.

### 2.2. Study Design

All measurements were implemented in a field study, i.e., at the children’s home under real-life condition. It was conducted during two measurement periods from June 2016 to November 2017 in the vicinity of Cologne-Bonn Airport. Cologne-Bonn Airport is an important passenger and cargo hub airport with the highest traffic densities at night mainly caused by freight traffic. About a quarter of daily flight movements take place between 10:00 p.m. and 6:00 a.m. [81]. Measurement sites were not exposed to major transportation or ambient noise sources other than air traffic. Bedroom windows of the participating children were not facing main roads or railways.

All children were studied for four consecutive nights. Measurements always started on a Wednesday. The time in bed of the study children corresponded to the age-appropriate sleep duration between 8 and 10 h [86]. Children slept with their usual window position in the bedroom. But they were asked not to change window position during the night. The first study night was not considered in the analysis as it served as adaptation to the polysomnographic devices used for objective sleep assessments [87]. To measure the short-term psychological effects of nocturnal aircraft noise, in the morning, children retrospectively rated the previous night with respect to their self-rated aircraft noise annoyance.

### 2.3. Acoustical Measurements

During all nights, sound pressure levels and sound files for the entire time in bed (=period between going to bed and getting up) were recorded inside the bedroom next to the children’s ear and in addition outside of the bedroom window. Recordings were performed by a Class-1 sound level meter XL2 from NTI Audio. The sound pressure levels were logged with an A-weighting and a slow-response (*L*_AS_) in the interval of one second. For subsequent noise identifying, wav-files were consistently recorded with 24 kHz sampling rate. The following acoustical parameters were determined based on the acoustical measurements inside the bedroom during each individual’s time in bed:

Number of aircraft (*N*_AC_) and number of aircraft overflights above threshold:

- *N*_AC_: Number of aircraft in total;
- *NAT*_30_–*NAT*_65_: Number of aircraft with a maximum level ≥ 30 dB, *NAT* was computed in 5 dB steps.

Maximum sound pressure levels (*L*_Amax_) and statistical metrics (*L*_X_):

- *L*_1_: Sound pressure level in dB which is exceeded during 1% of the AC time;
- *L*_10_: Sound pressure level in dB which is exceeded during 10% of the AC time;
- max *L*_Amax,AC_: Maximum sound pressure level of aircraft noise in dB (=maximum of the *L*_Amax_ of all overflights);
- mean *L*_Amax,AC_: Mean of the *L*_Amax_ values in dB of all single overflights per time in bed.

Energy equivalent sound pressure level:

- L_Aeq,AC_: A-weighted energy equivalent sound pressure level related to aircraft noise.

Aircraft to background noise ratio:

- *SNR*: Signal to noise ratio, ratio between *L*_Aeq,AC_ and the background sound pressure level, i.e., the *L*_Aeq_ for all sounds during bed time except aircraft sounds and noise produced by the participants themselves (e.g., coughing, snoring).

Time with aircraft noise:

- Total AC time: Overall time in seconds influenced by aircraft noise;

Statistical parameters of the aircraft noise metrics used in the present study are shown in Table 1.

### 2.4. Assessment of Annoyance and Non-Acoustical Factors

All surveys were linguistically adapted to the age of the children. Mostly standardized semantic five-point scales with un-/happy smileys as graphical anchors were applied. Children received a detailed explanation of the scales and questions.

Short-term annoyance from nocturnal aircraft noise exposure was measured retrospectively in the morning, 30 min after rising time, using survey software running on a netbook (Lime-Survey, Version 2.0, [88]). Information on relevant psychological factors which may have an influence on the relationship between aircraft noise and annoyance was surveyed in computer-assisted personal interviews with the children on the first study day. Besides demographical data, relevant psychological influence variables such as children’s noise sensitivity, attitudes towards air traffic, the application of measures to cope with the noise, and residential satisfaction were inquired in the interviews. In addition, further potential influence factors of the children’s annoyance response were assessed by a parent, such as the child’s estimated capability to adapt to the aircraft noise, socioeconomic status and housing situation of the family, or the parent’s own long-term aircraft noise annoyance during the past 12 months.

Long-term and short-term annoyance was measured by a question recommended by the International Commission on Biological Effects of Noise (ICBEN) [89,90]. For the purpose of comparability, instead of the original ICBEN scale, a similar standardized verbal five-point scale [91] was used which had already been applied in a prior aircraft noise study with adults around Cologne-Bonn Airport. This scale ranged from “1 = not” to “5 = very annoyed”. The answering categories were presented to the (random) half of the children in descending order and the other half in ascending order to control the influence of the order of scale categories on response.

### 2.5. Statistical Analysis

Logistic regression analysis (LRA) is a standard statistical procedure in noise effects research for the derivation of exposure–response curves [83,85,92,93,94]. Thus, LRA was used to determine the probability to be (highly and moderately) annoyed by nocturnal aircraft noise exposure as a function of the different acoustical and non-acoustical variables. The LRA is similar to linear regression analysis except that the predicted variable is binary, i.e., the dependent variable can be defined as an event that either takes place (1) or does not take place (0). In the present study, each child was examined repeatedly. Hence, the random effects model accounted for the non-independency of annoyance ratings by enclosing a random subject effect through a random intercept [95].

For LRA, a binary variable was determined. The Schultz criterion [96] regards individuals, whose ratings are distributed in the upper 25% to 30% of an annoyance scale (e.g., on a five-point scale the categories 4 and 5) as “highly annoyed”. However, a restriction on the percentage of persons who are highly annoyed by noise is questionable since the (likewise quantitatively important) proportion of those, whose noise annoyance falls into the intermediate range of the answering scale, is not considered. Therefore, we determined a binary variable via summarizing the response categories 3, 4, and 5 of the original annoyance five-point scale in order to differentiate between “moderate to high annoyance” (value 1) and “no or low annoyance” (value 0) represented by categories 1 and 2.

The modelling was done on the theoretical basis according to [60] and [27], under consideration of findings of noise-induced annoyance response in adults. Accordingly, potential acoustical and non-acoustical predictors of aircraft noise-induced annoyance in children were pre-selected. In addition to the aircraft noise metrics described in Table 1, the following non-acoustical parameters were considered in the modelling:

Variables obtained from the children sample:

a. Variables related to aircraft noise:
  - Evaluation of the residential area by means of an open question. The children’s answers were assessed by content analysis with respect to aircraft noise reference. Accordingly, answers were dichotomized (“1 = aircraft noise reference”, “0 = no reference”);
  - Perception of the sound level of aircraft in the home environment (“1 = not” to “5 = very loud”);
  - Long-term annoyance from chronic aircraft noise exposure of the previous 12 months (“1 = not” to “5 = very annoyed”);
  - Short-term annoyance from aircraft noise exposure of the previous day (“1 = not” to “5 = very annoyed”);
  - General attitude towards air traffic (“1 = very good” to “5 = very bad”);
  - Attitude towards air traffic with respect to its necessity, preventability, dan-ger/unsafety, health hazard, and fear of plane crashes (“1 = not true” to “5 = very true”);
  - Need for change in neighbourhood: “If you were a magician, what would you change in your neighbourhood, so that you and all the other children feel well here?”). Answers were dichotomized with respect to aircraft noise reference according to content analysis (“1 = aircraft noise reference”, “0 = no reference”);
  - Coping strategy in dealing with aircraft noise (e.g., closing windows or changing to a quieter room when aircraft are passing during homework is done) (“1 = never” to “5 = every day”).
b. Personal variables:
  - Age;
  - Gender;
  - Noise sensitivity (“1 = not” to “5 = very sensitive to noise”);
  - Presence of stressful events the day before (“1 = not” to “5 = very present”);
  - Fatigue in the evening before starting the measurements (“1 = very awake” to “5 = very tired”);
  - Fatigue in the morning (“1 = very awake” to “5 = very tired”);
  - Self-rated sleep quality (“1 = very good” to “5 = very bad”);

Variables obtained from the parents of the children sample.

a. Variables related to aircraft noise.
  - Children’s adaptation to chronic aircraft noise exposure (“1 = not” to “5 = very adapted”);
  - Parent’s long-term annoyance from chronic aircraft noise exposure of the previous 12 months (“1 = not” to “5 = very annoyed”).
b. Sociodemographic and personal variables.
  - Housing situation (i.e., residential characterization, house type, tenant or home ownership, occupancy in months);
  - Sociodemographic status of the main breadwinner in the family as measured by the Scheuch–Winkler-Index (SWI) [97].

For each of the acoustical and non-acoustical predictors, univariate analyses were carried out first. The selection of a prediction model for short-term aircraft noise-induced annoyance was achieved on the basis of expert experience and published literature, considering formal selection procedures. The decision as to whether a predictor was included was based on the *p*-value and the AIC (Akaike Information Criterion), which is an indicator of the goodness of statistical models [98]. Forward selection was carried on until there was no improvement with regard to the AIC. Only variables were included that showed at least a trend for an effect on annoyance (i.e., *p* ≤ 0.10). All factors were also tested for interactions and multicollinearity. 

Data were analyzed using SPSS 21 (IBM Statistics, Armonk, NY, USA). Final regression models are presented in tabular form.

## 3. Results

In the following section, the effect of nocturnal aircraft noise exposure and non-acoustical factors on short-term annoyance ratings in children are reported. First, we provided descriptive results on the nocturnal aircraft noise exposure and annoyance ratings (Section 3.1 and Section 3.2.1). In a second step, we investigated the association between a broad range of acoustical and non-acoustical variables, respectively, and the annoyance rating (Section 3.2.2). Subsequently, exposure–response models and curves were derived showing the association between the nocturnal aircraft noise exposure (described by the *N*_AC_ and the *L*_Aeq,AC_, respectively) and annoyance and considering the effect of relevant non-acoustical factors (Section 3.2.2). As a final step, we compared the exposure–response curves (Section 3.3.1) as well as the effect size of the predictors (Section 3.3.2) between children and adults.

### 3.1. Aircraft Noise Exposure during the Study Nights

Forty-eight children of the original sample were included in the data analysis. Data of three participants could not be considered due to missing data mainly in the personal interview. In total, data from 134 nights were used. Aircraft noise exposure assessed inside the bedroom at the sleeper’s ear during these study nights is summarized in Table 1. For the calculation of most of the acoustical parameters, all aircraft noise events recorded during the time in bed were considered, including those overlapping with sounds from other sources, e.g., road and rail noise events or sounds from the participant. An exception was made for the parameters *L*_1_ and *L*_10_ as well as the highest and the averaged maximum noise levels, as these four parameters describe different kinds of maximum levels of aircraft noise events and, thus, can easily be biased by other loud (indoor) ambient sounds. For the calculation of these parameters, only aircraft noise events were included that did not overlap with other ambient sounds.

### 3.2. Short-Term Annoyance from Nocturnal Aircraft Noise Exposure in Children

#### 3.2.1. Descriptive Statistics

**Figure 1:**
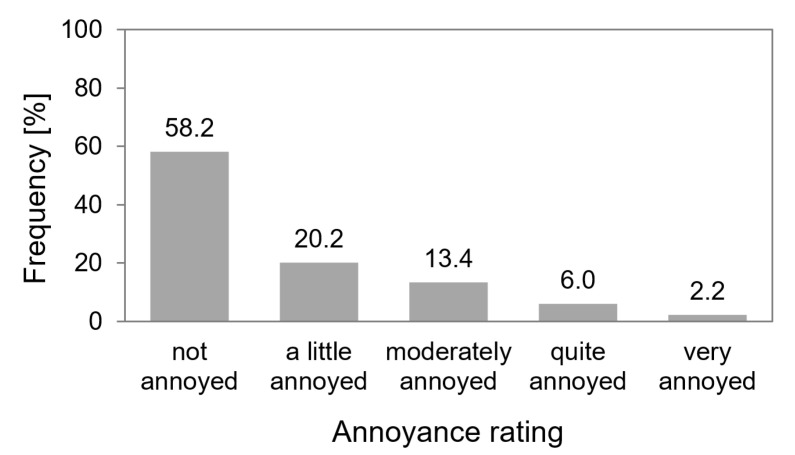
Percentage distribution of aircraft noise-induced short-term annoyance due to exposure of the previous night (*N* = 48 subjects, 134 nights).

Figure 1 shows the percentage frequency distribution of the answer to the question “How much have you been disturbed or bothered by aircraft noise of the last night?” by using a five-point answering scale. As shown by Figure 1, 78.4% of the children felt “not” or “a little” (categories 1 and 2 of the original scale) annoyed. 21.6% of the ratings were in the range of “moderately and very” annoyed (categories 3 to 5).

#### 3.2.2. Exposure–Response Models

The univariate analyses on acoustical parameters indicated that none of the aircraft noise metrics proved statistically significant (see Supplementary Table S1). Table 2 presents the results of the univariate analyses for those non-acoustical predictors that were significant at least on a trend level (*p* ≤ 0.10. Since the final *p*-values of the regression parameters may be biased due to the model selection process [99], OR and the 95% CI are also presented. The effect of a predictor is considered significant if the OR is different from 1 and the 95% CI is not covering 1.

As a first predictor in the null model, noise sensitivity was included as substantial evidence exists for its impact on transportation noise-induced annoyance [72,77]. In a second step, attitudes were regarded as potential predictor, since attitudes towards air traffic are also viewed as important determinants of noise annoyance [72,77]. Attitude variables (e.g., AC are dangerous, AC are useful, fear of plane crashes) were added one by one to the modelling. Afterwards, additional non-acoustical factors were tested for a significant contribution to annoyance and an improvement of the model fit. The best model based on AIC included the variables “noise sensitivity” and “AC are dangerous” and the application of measure to cope with the noise, short: “coping” (Table 3).

In the next step, acoustical predictors were integrated into the annoyance model LR1. Since the univariate regression analyses did not show any significant effects of the examined aircraft noise parameters, two established noise metrics were selected: the number of aircraft (*N*_AC_) and the energy equivalent sound pressure level for aircraft noise during the time in bed (*L*_Aeq,AC_). However, neither of the two metrics proved significant in combination with model LR1. As shown by Table 4 and Table 5, only noise sensitivity retained its significant increasing influence on children’s short-term annoyance (LR2: OR = 2.171, 95% CI 1.142–4.127, *p* = 0.018; LR3: OR = 2.204, 95% CI 1.153–4.211, *p* = 0.017). The attitude variable “AC are dangerous” and coping behavior were significant in tendency (*p* ≤ 0.10) (LR2: OR = 1.721, 95% CI 0.957–3.096, *p* = 0.069; LR3: OR = 1.739, 95% CI 0.971–3.113, *p* = 0.062).

**Figure 2:**
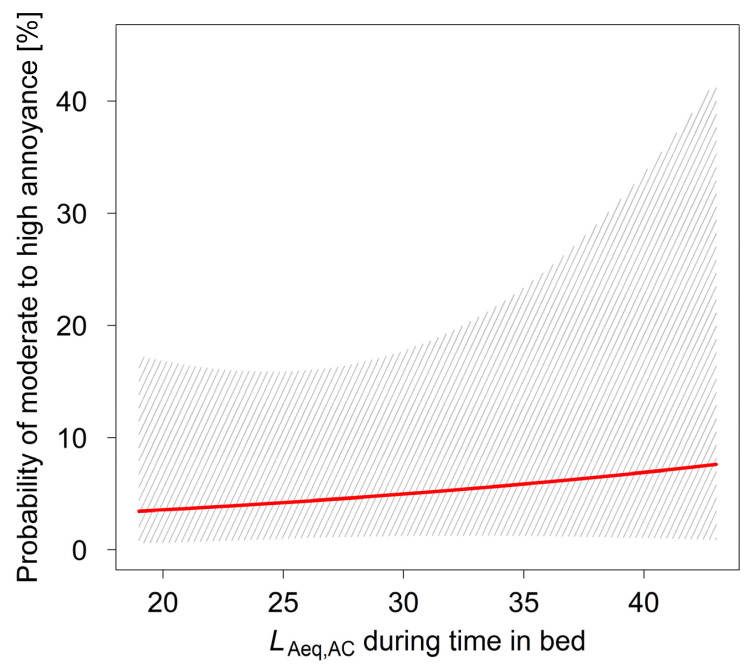
Probability for annoyance (categories ≥ 3) by aircraft noise of the previous night as predicted by model LR2 depending on the energy equivalent noise level for aircraft noise (*L*_Aeq,AC_) during time in bed. The hatched area shows the 95% CI.

**Figure 3:**
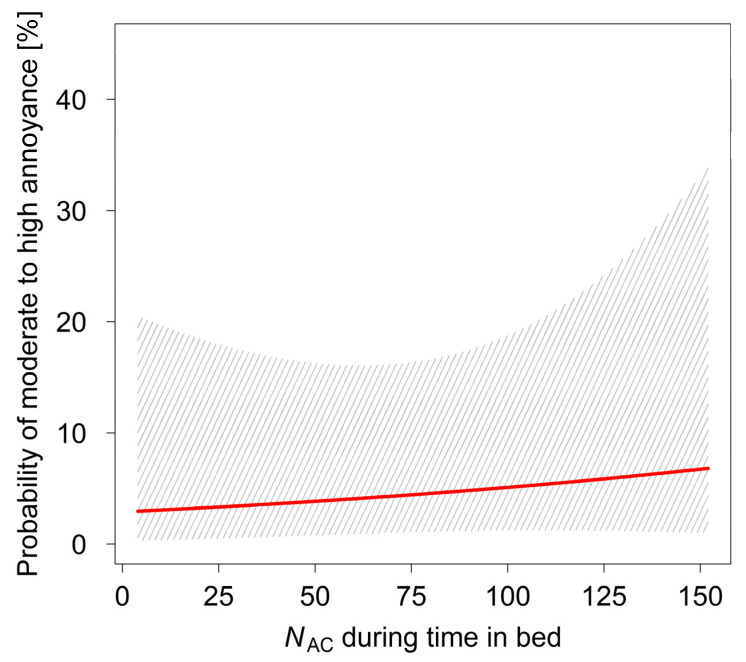
Probability for annoyance (categories ≥ 3) by aircraft noise of the previous night as predicted by model LR3 depending on the number of aircraft (*N*_AC_) during time in bed. The hatched area shows the 95% CI.

Figure 2 and Figure 3 depict the probability for annoyance in children depending on the energy equivalent noise level *L*_Aeq,AC_ (model LR2) and the number of nocturnal aircraft (model LR3) during time in bed, respectively. There was neither a significant increase of annoyance probability with rising *L*_Aeq,AC_ nor with growing number of nocturnal overflights. For both figures, the non-acoustical variables in the models were set to the median in the sample (i.e., noise sensitivity = 2, AC are dangerous = 2, coping = 1).

### 3.3. Comparison of Aircraft Noise-Induced Short-Term Annoyance in Children and Adults

#### 3.3.1. Exposure–Response Models

**Figure 4:**
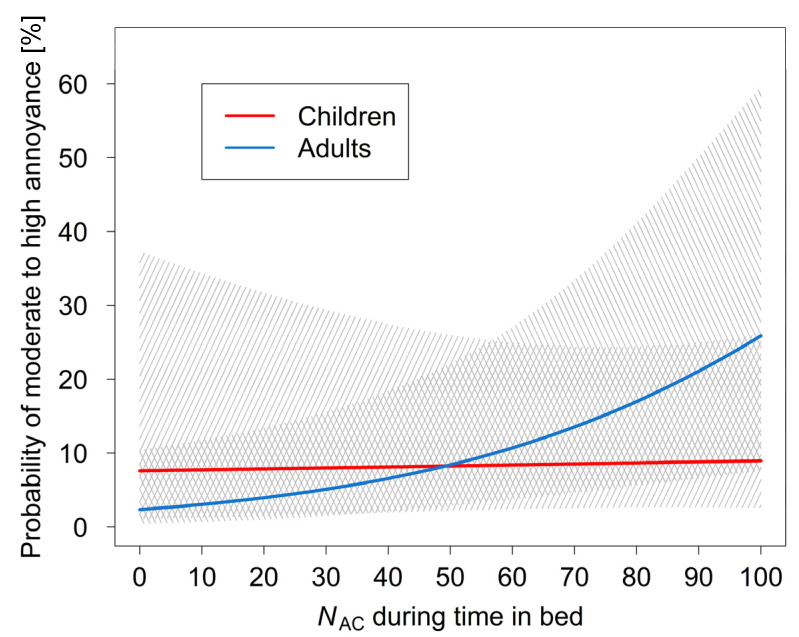
Probability for annoyance (categories ≥ 3) by aircraft noise of the previous night as predicted by model LR4 depending on *N*_AC_ with corresponding 95% CI (hatched area).

From 2001 to 2002 the DLR carried out the field study STRAIN with 64 adult residents aged 19 to 61 years (35 female) in the surrounding of Cologne-Bonn Airport. Study areas, design and methods [85,100] corresponded to those in the present study in children. Data of the present study in children and the STRAIN study were compared via applying the exposure–response model for short-term annoyance from nocturnal aircraft noise that was found in the STRAIN study [85]. For this purpose, the two data sets were pooled. In order to make the data sets comparable, a random sample of 48 subjects was selected from the STRAIN study including only measurements from the second to the fourth night. Table 6 summarizes the regression-based comparison of short-term aircraft noise annoyance in the two studies based on the STRAIN model [85]. As evidenced by the indicator variable “study”, the two studies differed significantly with respect to short-term noise annoyance (LR4: OR = 0.117, 95% CI 0.017–0.808, *p* = 0.030) even when controlled for the effects of age and adaptation to aircraft noise. The exposure–response curve is depicted in Figure 4. Adaptation to chronic aircraft noise exposure was set to 4. Medians for age were 8.5 years (children) and 40 years (adults). Annoyance probability in the STRAIN study was significantly higher than in the present children’s study. For adults, but not for children, the probability to be moderately to highly annoyed increased with rising numbers of aircraft flyovers during the time in bed.

#### 3.3.2. Comparison of the Predictor’s Effect Sizes by Using Forest Plots

**Figure 5:**
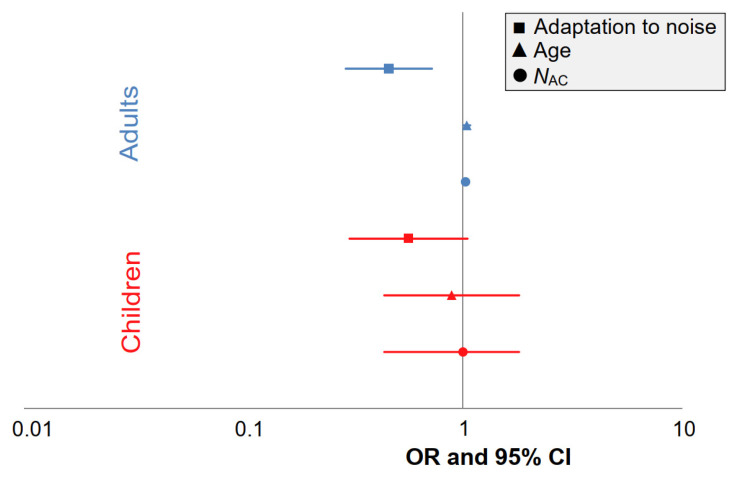
Forest plot depicting the OR and 95% CI for children (*N* = 48, red) and adults from the STRAIN study (*N* = 48, blue) for the acoustical and non-acoustical predictors of annoyance in model LR4.

To compare the influence of the predictors between children and adults, the annoyance model [85] originally developed for the STRAIN study were separately recalculated for children’s and adults’ data. Similar to the pooled model, only 48 subjects from the STRAIN study with three measurement nights were considered. None of the acoustical and non-acoustical predictors in model LR4 reached statistical significance in a children-only model. For the STRAIN data, the acoustical aircraft noise parameter *N*_AC_ (OR = 1.025, 95% CI 1.005–1.045, *p* = 0.013,) as well as age (OR = 1.051, 95% CI 1.003–1.101, *p* = 0.037) were statistically significant. However, as illustrated in Figure 5, the direction of effects in children and adults, with the exception of age, was the same. With respect to the acoustical predictors, there were no marked differences between children and adults in terms of OR, but the confidence intervals were much larger in children.

## 4. Discussion

Studies dedicated to the short-term psychological effects of nocturnal aircraft noise exposure on children are rare. So far, there have been hardly any exposure–response models describing the relationship between aircraft noise exposure and the resulting short-term annoyance reactions in children. The present study was intended to close this research gap by means of a field study conducted around Cologne-Bonn Airport with 51 children aged 8 to 10 years. In order to rule out answer biases in the recall of noise (annoyance situations), annoyance was assessed every morning in the home environment and referring to the past night instead of simply asking for retrospective ratings for the past months. We developed new exposure–response models to predict aircraft noise-induced short-term annoyance considering a broad range of acoustical and non-acoustical factors as potential predictors of annoyance. The modelling assumed that—similar to adults—both acoustical and non-acoustical factors affect children’s annoyance. The contribution of both kinds of determinants to both short-term and long-term noise annoyance has been extensively validated in adults [13,39,71,72,73,75,76,77,80,81,83,85] whereas only few studies have been done in children [39,84].

Exposure–response models for the aircraft noise-induced short-term annoyance in children were calculated by means of random effects logistic regression considering repeated measurements for the same individual. Pre-selection of potential acoustical and non-acoustical predictors for the new children’s annoyance model was based on literature research. Univariate analyses indicated that none of the aircraft noise metrics proved statistically significant. It is possible that the current sample size (*N* = 51, 134 nights), which was derived from similar studies in adults, was not large enough to detect more subtle effects of aircraft noise in children. However, noise sensitivity [18,73,77,78,79] and attitude variables [13,77,80,81,82,83] were significant psychological determinants. After pre-selection, the modelling was performed by using literature-based forward selection. The best model (LR1) according to the AIC integrated the self-rated noise sensitivity, the attitude variable “AC are dangerous” and the children’s coping behavior. Integration of established aircraft noise metrics such as *L*_Aeq,AC_ (model LR2) and *N*_AC_ (model LR3), however, did not improve the quality of the final (non-acoustical) annoyance model.

From the present results, it can be concluded that children’s short-term annoyance response to night-time aircraft noise was primarily influenced by non-acoustical variables rather than the noise exposure itself. In particular, the noise sensitivity played a central role. According to Stansfeld and Clark [43] children may be more sensitive to noise than adults because they are in an important phase of physical and mental development. Van Kamp and Davies [22] assumed that noise sensitivity-related effects (in children) may be an expression of a more general vulnerability, which could originate from psychological and/or physiological factors. Children may also cope with noise in a different (possibly less effective) way than adults [28,30,31,43,60,100]. For instance, Bartels et al. [81] found an increasing effect of coping on long-term annoyance in adult airport residents, i.e., carrying out coping measures was related to a higher aircraft noise annoyance. The range of strategies that children employ to cope with noise exposure depends on the perceived amount of control they have over the noise source. However, children are assumed to be less capable of assessing threat from environmental stressors and thus have very little control over threatening situations [60]. As a consequence, children may not yet have developed suitable coping strategies [22,43]. Thus, children may not be more vulnerable to noise effects per se, but may be more at risk due to less-developed coping strategies and susceptible developmental processes.

The present cross-sectional comparison that was based on a model originally developed by Quehl and Basner [85] in the framework of the STRAIN study revealed that annoyance probability was significantly higher in adults than in children. An exposure–response curve revealed that at lower exposure, the probability for moderate to high annoyance was similar between children and adults whilst the probability for annoyance at higher exposure was lower in children. The result is reminiscent of previous studies on long-term annoyance from chronic aircraft noise exposure [22,42,67,68,70] indicating that children were less annoyed than adults. Moreover, we found that in adults but not in children annoyance probability depended on aircraft noise metrics. Accordingly, the percentage of moderately to highly annoyed adults grew with the frequency of overflights, whereas such a relationship was absent in children. Children may have fewer experiences to compare with, whereas adults more likely have lived in different places with different degrees of noise exposure that have shaped their experience and, thus, their expectation or reference level of environmental noise exposure. Moreover, the lesser role of acoustical factors in children may be due to their lower probability to awaken from aircraft noise during the night [101]. Thus, the experience of noise-induced sleep disruption—a prominent factor in adults—may contribute less to the overall perception of aircraft noise of children; other experiences, e.g., aircraft-noise situations during the daytime, most likely have a greater influence on children’s individual attitudes and behavior and, thus, on their annoyance ratings. This assumption is supported by the significant positive associations among the short-term annoyance during the past night, the annoyance reported for the preceding day, and long-term annoyance of the past 12 months. However, a comprehensive examination of such interrelationships requires longitudinal data and, hence, cannot be examined here.

The present work shows that the extent and etiology of annoyance due to transport noise can differ substantially among subgroups of the population. The reason for the lower annoyance probability in children is not yet fully understood and deserves more research. In general, there is a need to focus on transport noise effects in vulnerable people, of which children and adolescents represent important but understudied subgroups. Although children seem to be less annoyed by aircraft noise, they still need to be considered vulnerable in that continued noise exposure may interfere with their development. Future research on transport noise will benefit from establishing subgroup-specific exposure–response relationships for various domains of health, hence enabling to derive adequate noise protection for those that are vulnerable.

## 5. Conclusions

In contrast to adults, children’s short-term annoyance response to aircraft noise exposure during the previous night is not determined by acoustical parameters primarily. Personal factors play a more important role, including noise sensitivity, the use of coping measures, the attitude that aircraft are dangerous, as well as the capability to adapt to the noise. Annoyance ratings of children and adults differ depending on the level of noise exposure (represented by the number of aircraft during the time in bed); whereas, at lower exposure the probability of moderate to high annoyance is similar for the two groups, and children show a lower probability than adults at higher exposure. With regard to short-term annoyance due to aircraft noise, children do not seem to be a group at higher risk, but this must not be generalized to other noise-related health effects.

## Figures and Tables

**Table 1:** Aircraft noise parameters averaged across study nights and subjects (*N* = 48) based on the indoor measurements related to the individual bed times. Mean values (*M*) and standard deviation (SD) were calculated across study nights and across subjects.

Parameter	Minimum	Maximum	*M*	SD
*N*_AC_	4.0	152.0	71.5	25.1
*NAT*_30_	0.0	125.0	56.6	27.9
*NAT*_35_	0.0	119.0	43.9	29.3
*NAT*_40_	0.0	114.0	28.3	27.2
*NAT*_45_	0.0	113.0	16.5	23.7
*NAT*_50_	0.0	106.0	8.4	18.5
*NAT*_55_	0.0	90.0	3.4	11.5
*NAT*_60_	0.0	20.0	0.6	2.5
*NAT*_65_	0.0	4.0	0.1	0.4
*L*_1_	18.0	56.4	35.2	7.7
*L*_10_	17.5	38.5	23.0	4.5
max *L*_Amax,AC_	28.7	71.1	47.4	7.9
mean *L*_Amax,AC_	24.4	58.9	39.4	7.2
*L*_Aeq,AC_	18.5	43.3	26.3	5.2
*SNR*	0.49	1.0	0.9	0.8
Total AC time [s]	195.9	10,537.7	4841.7	2045.7

All acoustical parameters were calculated for the time in bed (=the period between going to bed and getting up).

**Table 2:** Univariate logistic regression analyses with random effects of the non-acoustical predictors for aircraft noise-induced short-term annoyance at night in children. Odds Ratios (OR) with 95% Confidence Intervals (CI) and *p*-values. Illustrated predictor variables were measured using five-point scales.

	OR	CI	*p*-Value
Annoyance from aircraft noise exposure during daytime of the previous day	2.838	1.622–4.965	0.000
Self-rated sleep quality	2.267	1.385–3.712	0.001
Long-term annoyance from chronic aircraft noise exposure of the previous 12 months	2.456	1.360–4.436	0.003
Noise sensitivity assessed by the child	2.356	1.328–4.178	0.004
AC are dangerous	1.880	1.108–3.191	0.020
Fatigue in the morning	1.765	1.071–2.910	0.026
Coping	1.556	1.044–2.318	0.030
Fear of plane crashes	1.726	1.075–2.769	0.024
AC are useful	0.634	0.402–1.000	0.050
Children’s adaptation to chronic aircraft noise exposure assessed by parents	0.551	0.315–0.962	0.036

**Table 3:** Multiple logistic regression model LR1 with random effects for the prediction of aircraft noise-induced short-term annoyance at night in children. Adjusted Odds Ratios (OR) with 95% Confidence Intervals (CI) and *p*-values, AIC = 652.

	Estimate	Standard Error	OR	CI	*p*-Value
Intercept	−5.437	1.257	0.004	0.000–0.052	0.000
Noise sensitivity	0.741	0.313	2.099	1.131–3.897	0.019
AC dangerous	0.541	0.291	1.718	0.965–3.058	0.066
Coping	0.363	0.215	1.438	0.939–2.202	0.094

**Table 4:** Multiple logistic regression model LR2 with random effects for the prediction of aircraft noise-induced short-term annoyance of children. Acoustical variable: *L*_Aeq,AC_ during time in bed. Adjusted Odds Ratios (OR) with 95% Confidence Intervals (CI) and *p*-values, AIC = 659.

	Estimate	Standard Error	OR	CI	*p*-Value
Intercept	−6.239	2.037	0.002	0.000–0.110	0.003
*L*_Aeq,AC_	0.026	0.052	1.026	0.926–1.138	0.617
Noise sensitivity	0.775	0.325	2.171	1.142–4.127	0.018
AC dangerous	0.543	0.297	1.721	0.957–3.096	0.069
Coping	0.370	0.219	1.448	0.939–2.234	0.093

**Table 5:** Multiple logistic regression model LR3 with random effects for the prediction of aircraft noise-induced short-term annoyance at night of children. Acoustical variable: N_AC_ during time in bed_._ Adjusted Odds Ratios (OR) with 95% Confidence Intervals (CI) and *p*-values, AIC = 661.

	Estimate	Standard Error	OR	CI	*p*-Value
Intercept	−5.921	1.543	0.003	0.000–0.057	0.000
*N*_AC_	0.005	0.009	1.005	0.987–1.023	0.586
Noise sensitivity	0.790	0.327	2.204	1.153–4.211	0.017
AC dangerous	0.553	0.294	1.739	0.971–3.113	0.062
Coping	0.350	0.220	1.419	0.919–2.192	0.114

**Table 6:** Logistic regression model LR4 with random effects for the prediction of aircraft noise-induced short-term annoyance at night measured in the children’s and STRAIN (adults’) studies, with *N*_AC_ during time in bed, age and the adaptation to aircraft noise as independent variables (AIC = 1349).

	Estimate	Standard Error	OR	CI	*p*-Value
Intercept	−0.708	0.821	0.493	0.098–2.479	0.389
*N*_AC_	0.010	0.006	1.010	0.998–1.022	0.091
Age	0.043	0.024	1.044	0.993–1.098	0.089
Adaptation to aircraft noise	−0.509	0.191	0.601	0.412–0.877	0.009
Study ^1^	−2.146	0.892	0.117	0.017–0.808	0.030

^1^ Reference: Adult sample of the STRAIN study.

## Data Availability

Not applicable.

## Supplementary Materials

The following are available online at https://www.mdpi.com/article/10.3390/ijerph18136959/s1, Table S1: Univariate logistic regression analyses with random effects of several acoustical predictors for aircraft noise-induced short-term annoyance at night in children. Odds Ratios (OR) with 95% Confidence Intervals (CI) and *p*-values. (*N* = 134 nights, 48 subjects).
