# Supplementary material for: Effects of Nocturnal Aircraft Noise and Non-Acoustical Factors on Short-Term Annoyance in Primary School Children
Source: Int J Environ Res Public Health. 2021 Jun 29;18(13):6959. doi: 10.3390/ijerph18136959 (PMC8297142; doi:10.3390/ijerph18136959)
Supplement: Supplementary file 1 [file ijerph-18-06959-s001.zip › ijerph-1263100-supplementary.pdf]

Supplementary Table S1. Univariate logistic regression analyses with random effects of several acoustical predictors for aircraft noise-induced short-term annoyance at night in children. Odds Ratios (OR) with 95 % Confidence Intervals (CI) and p-values. (N = 134 nights, 48 subjects).

	**OR**	**CI**	***p*-Value**
N_{AC}	0.999	0.983-1.015	.897
NAT_{30}	0.998	0.982-1.014	.766
NAT_{35}	0.997	0.981-1.014	.754
NAT_{40}	1.000	0.982-1.017	.966
NAT_{45}	0.997	0.977-1.017	.756
NAT_{50}	0.998	0.971-1.026	.880
NAT_{55}	0.994	0.947-1.043	.792
L_1	1.000	0.940-1.064	.992
L_{10}	0.984	0.882-1.098	.770
max $L_{Amax,AC}$	1.000	0.945-1.057	.987
mean $L_{Amax,AC}$	1.004	0.939-1.073	.910
$L_{Aeq,AC}$	0.998	0.907-1.097	.960
SNR	1.314	0.013-135.317	.907
Total AC time [sec]	1.000	1.000-1.000	.603

Note. The effect of NAT_{60} to NAT_{75} is not reported because of a too low number of valid data ($n \leq 22$).
